# Digital Detectives: Websleuthing Reduces Eyewitness Identification Accuracy in Police Lineups

**DOI:** 10.3389/fpsyg.2021.640513

**Published:** 2021-04-15

**Authors:** Camilla Elphick, Richard Philpot, Min Zhang, Avelie Stuart, Graham Pike, Ailsa Strathie, Catriona Havard, Zoe Walkington, Lara A. Frumkin, Mark Levine, Blaine A. Price, Arosha K. Bandara, Bashar Nuseibeh

**Affiliations:** ^1^School of Psychology and Counselling, The Open University, Milton Keynes, United Kingdom; ^2^Department of Psychology, Lancaster University, Lancaster, United Kingdom; ^3^School of Computing & Communications, The Open University, Milton Keynes, United Kingdom; ^4^Department of Psychology, University of Exeter, Exeter, United Kingdom; ^5^Lero, University of Limerick, Limerick, Ireland

**Keywords:** digital detective, websleuth, eyewitness, lineup identification, police lineup, social media, post-event information

## Abstract

Eyewitnesses to crimes sometimes search for a culprit on social media before viewing a police lineup, but it is not known whether this affects subsequent lineup identification accuracy. The present online study was conducted to address this. Two hundred and eighty-five participants viewed a mock crime video, and after a 15–20 min delay either (i) viewed a mock social media site including the culprit, (ii) viewed a mock social media site including a lookalike, or (iii) completed a filler task. A week later, participants made an identification from a photo lineup. It was predicted that searching for a culprit on social media containing the lookalike (rather than the culprit) would reduce lineup identification accuracy. There was a significant association between social media exposure and lineup accuracy for the Target Present lineup (30% more of the participants who saw the lookalike on social media failed to positively identify the culprit than participants in the other conditions), but for the Target Absent lineup (which also included the lookalike) there was no significant association with lineup identification accuracy. The results suggest that if an eyewitness sees a lookalike (where they are expecting to see the culprit) when conducting a self-directed search on social media, they are less likely to subsequently identify the culprit in the formal ID procedure.

“Websleuthing” describes amateur online crime investigations (e.g., Yardley et al., 2018) such as “Facebook identifications” (Mack and Sampson, 2013) or “crowdsourcing” [Estellés-Arolas, (2020)], which is the practice of engaging a crowd *via* digital technologies to achieve a common goal. Websleuthing has become widespread on crime-solving can both contribute to, and hamper, police investigations, as described below.

Through crowdsourcing, individual expertise and digital technologies can complement law enforcement approaches [Estellés-Arolas, (2020)]. Social media users have helped to solve cases, found or verified evidentiary material^1^, extended community policing^2^, and raised awareness of missing people^3^. Indeed, following an attack in Philadelphia, police released video footage and Twitter users were able to track down the perpetrators, resulting in several positive identifications (Shaw, 2014).

There is additional evidence of positive outcomes from police research. In a survey of police officers in the US (NCJRS, 2013), 80.4% responded that social media had helped to solve crimes, while Kim et al. (2017) found that 89% of participants from Law Enforcement agencies claimed to use social media for disseminating information, and 58% had contacted social media companies for evidence. Following interviews of police investigators in Norway, Denmark, and Sweden, Rønn et al. (2020) concluded that social media should be part of investigative police work, as it provides information that is not available in police files.

However, Pantumsinchai (2018) argued that collective intelligence during unfolding events can be problematic. For instance, as an official police investigation was taking place into the Boston Marathon bombing in 2013, “Reddit” users conducted their own investigation (Nhan et al., 2015) and named innocent people who had not been involved (Lee, 2013; Nhan et al., 2015; see also Lally, 2017). Reddit is a social media site with posts known as “subreddits.” In 2012, a subreddit called the Reddit Bureau of Investigation (RBI) was launched to solve crimes (Myles et al., 2020). Similarly, after the Erawan Shrine bombing in 2015, websleuthing resulted in doxing (seeking and publishing of private or identifying information online) and shaming, without resulting in a positive identification (Pantumsinchai, 2018). Given the negative consequences of websleuthing, Yardley et al. (2018) concluded that it may just be a version of true crime “infotainment.”

Websleuthing also falls outside existing regulatory frameworks, so social media identifications can create complications at trial. This has happened in the US (e.g., *State of Oregon v. Soza, 2008*^4^—police showed a witness a MySpace image of Soza after they had been unable to identify him in a lineup. This led to an appeal, but the conviction held.); the UK (e.g., *R v McCullough, 2011*^5^—lineup identification evidence was deemed unreliable at appeal as it had come after a self-directed Facebook identification, but the conviction held. *; R v Alexander and McGill, 2012*^6^—police were criticized at appeal, after failing to disclose details of a self-directed Facebook identification that preceded a formal lineup procedure, but the convictions stood.); and in Australia (e.g., *R v Benfield, 2015*^7^—formal identification evidence that came after a Facebook search was deemed inadmissible). The Police and Criminal Evidence Act (PACE) guidelines have since been updated to include social media evidence in England and Wales, although are aimed at situations when, “…for the purpose of identifying and tracing suspects, films and photographs of incidents or other images are… shown to the public (which may include police officers and police staff as well as members of the public) through the national or local media or any social media networking site…” (Police and Criminal Evidence Act 1984, 2017, Code D, Section 3, Part C, Paragraph 3.38). However, the effects of websleuthing on criminal investigations have seldom been tested empirically with regards to eyewitness identifications. This is surprising, considering that the leading cause of wrongful convictions in the US is related to eyewitness identifications, which are a contributing factor in 75% of DNA exonerations (Innocence Project, 2008). Comparable data is not available in the UK. Injustices may also have been compounded by the rise in social media, as searching for a suspect online (Wilson, 2013) could potentially undermine police lineup safeguards.

Support for this suggestion comes from studies that have explored the effects of other forms of post-event information on eyewitness identification. Deffenbacher et al. (2006) meta-analysis showed that exposure to mugshot images reduced subsequent correct identifications. Similarly, Valentine et al. (2012) found that street identifications can bias subsequent identification decisions. This is supported by a field study in which 84% of suspects identified in the street were identified in a subsequent lineup (Davis et al., 2015). One interpretation of the effect is a “commitment effect” (Godfrey and Clark, 2010; Valentine et al., 2012; Davis et al., 2015), as witnesses who identify an individual in one identification procedure are more likely to select the same individual in a second identification procedure, regardless of the accuracy of the first identification (Blunt and McAllister, 2009). Another interpretation is that a form of unconscious transference is at work (Deffenbacher et al., 2006), where the face seen in the post-event information (e.g., a mugshot) makes the face of the perpetrator in the witness's memory inaccessible.

As well as mugshots and street identifications, research has also explored the possible effect that constructing a facial composite may have on subsequent lineup accuracy. This has real-world importance because 27% of eyewitness misidentifications reported by the Innocence Project (n.d.) involved facial composite sketches. However, although some studies have shown impaired identification performance following composite construction (e.g., Wells et al., 2005), others have found that identification accuracy improved (e.g., Davis et al., 2014) with most research tending to find no effect (e.g., Pike G. E. et al., 2019; Pike G. et al., 2019). A meta-analysis of this research revealed no significant negative effects of composite *construction* (Tredoux et al., 2020), although it is possible that *exposure* to a composite created by someone else may have a negative effect if the suspect and composite image share the same misleading feature, or either a positive or no effect if the composite is a more accurate representation (Sporer et al., 2020).

The issues related to composite construction, street identifications, and mugshot inspections are relevant to social media investigations, as making a self-directed culprit search online could also bias a subsequent lineup identification. The study presented here was designed to address a particular real-world issue. After witnessing a crime, some people use social media to seek the culprit. If the witness finds someone that they think is the culprit on social media, they can go to the police with this information. Under UK PACE guidelines the police must still conduct a formal lineup, which will contain the suspect the witness identified on social media (whether they are the culprit, or an innocent lookalike). If a witness searches for the culprit on social media, finds a plausible “lookalike” individual and reports them to the police, the witness might then either “unconsciously transfer” or “commit” to that face such that they would identify this individual in a subsequent lineup. Of course, if the witness finds an image of the culprit on social media, then the same commitment effect would make them more likely to select that face in a lineup, although the identification might be more based on the more recent viewing of social media than their actual memory of the crime. In addition, a witness who sees a lookalike on social media but then sees the *culprit* in a lineup, might fail to identify them if their memory had been contaminated by the lookalike. To address this issue, the present study aimed to investigate the effect of searching for a culprit on social media on subsequent lineup identification accuracy. We predicted that if an eyewitness sees a “lookalike” when conducting a social media search, they are less likely to subsequently identify the culprit in the formal ID procedure (Deffenbacher et al., 2006).

## Methods

### Participants

We based our sample size on power considerations derived from comparable research (Havard et al., under review) that indicates a large effect size (Cramer's *V* = 0.29, *df* = 4) (Cohen, 1988). Given the inflated estimates of effect sizes in the literature (e.g., Open Science Collaboration, 2015), we aim to detect a more conservative, medium-large effect. Two a-priori power analyses, corresponding to the two conditions (TP and TA), were carried out in G^*^Power (Faul et al., 2007). The power analysis for the TP condition estimated a required sample size of 142 participants to detect a medium-large effect with a statistical power of 0.90, and α = 0.05 (required Cramer's *V* = 0.23, Cohen's *W* = 0.33, with *df* = 4) (Cohen, 1988). The power analysis for the TA condition required a sample size of 132 (required Cramer's *V* = 0.31, Cohen's *W* = 0.31, with *df* = 2) (Cohen, 1988).

We sampled 285 participants with access to the internet (149 in the Target Present (TP) condition, and 136 in the Target Absent (TA) condition) (see Table 1). The sample included 11 more participants than required, as the two experimental sessions were a week apart, so we could not be certain how many participants that completed session 1 would complete session 2. They were recruited *via* social media and university research participation sites. Of these, 273 provided their age (*M* = 28.52; *SD* = 14.85), and 277 provided their gender: 231 identified as female; 44 as male; one as androgynous; and one as non-binary. Two hundred and twenty-eight provided their ethnicity: 171 identified as Caucasian; 34 as Asian (14 of which identified as Far East Asian while four identified as South Asian); four as Mixed Race; two as Black; two as Mediterranean; and one as Arab; one as Hispanic; and one as Jewish.

**Table 1:** Experimental conditions, design, and number of participants.

**FriendFace condition**	**Culprit**	**Culprit**	**Lookalike**	**Lookalike**	**Control**	**Control**
Watched crime video	Yes	Yes	Yes	Yes	Yes	Yes
Completed survey	Yes	Yes	Yes	Yes	Yes	Yes
Viewed FriendFace	Yes	Yes	Yes	Yes	No	No
One week delay	Yes	Yes	Yes	Yes	Yes	Yes
Lineup type	Present	Absent	Present	Absent	Present	Absent
Number of participants	51	50	45	42	53	44

Participants did not receive compensation for their participation.

### Design

The experiment employed a 3 × 2 between participant design. The first independent variable was self-directed *social media* search, with three levels: Culprit (social media containing the culprit), Lookalike (social media containing a lookalike), Control (no social media exposure). The second independent variable was *lineup type*, with two levels: TP (culprit in the lineup) and TA (lookalike in the lineup). The first dependent variable was *identification accuracy*. In the TP lineups, participants could make one of three responses: a correct identification, a misidentification, or an incorrect rejection. In TA lineups, participants could make one of two responses: a correct rejection, or a misidentification. Data from the TP and TA lineups were analyzed separately. The second dependent variable was confidence, measured on a 7-point scale from 1 (very unsure) to 7 (very sure).

### Materials

The experiment was built as a series of simple webpages (hosted on the research group website), consisting of two sessions.

Session one presented an information page, a consent page, a mock crime video, a 15–20 min survey (exploring online police/citizen communication), which acted as a filler task and informed the development of measures for a police/citizen app scoping study. Participants then either viewed a simulated social media site (FriendFace) or took part in an alternative activity [Ministry of Sharing, (n.d.)]. The latter tested whether participants share too much information online.

Session two consisted of a photo lineup display consisting of six simultaneously presented faces and two response boxes. The lineup was created using PROMAT Video Identification Parade Software. Photos showed the head and shoulders of each individual against a green PROMAT background, photographed in the same lighting conditions. The target present display contained an image of the culprit, while the target absent display contained an image of the lookalike. These were placed in the same position in their display. As participants saw the lineup online, on personal devices, we could not control the size or color saturation of the images between participants. The method described by Tredoux (1998) was used to calculate the effective size of the target present lineup. Seventy-three participants made a forced-choice selection from the lineup based on a verbal description of the target and analysis showed an effective size (E) of 4.05, with two of the foils being selected more frequently than the target.

The mock crime video was created with a Caucasian male actor as the target (the culprit). The video shows two women seated at a bar while the culprit walks in and steals a handbag hanging from a stool. The culprit then leaves the bar. The film lasts ~30 s, and the culprit is visible from multiple viewpoints and close up during the film.

FriendFace was created to mimic a social network site and was populated with content relating to the community event, where the mock crime took place. The home page contained event photos, and names and thumbnail pictures of 14 people who had indicated interest in attending (seven men and seven women). Thumbnails consisted of a color close up of the head and shoulders, taken in natural settings. By clicking on the thumbnails, participants could view the “profiles” of each potential attendee, including a larger version of the thumbnail, and two additional images of them, where their face could be seen from another viewpoint and in different lighting conditions (see Supplementary Material). For the experimental manipulation, two versions of a profile page were created from actors matched according to age, build, and general appearance. One version contained photos of the culprit but not the lookalike (culprit condition), and the other contained photos of the lookalike but not the culprit (lookalike condition). Photos were provided by each individual on the FriendFace site (including the culprit and the lookalike) from their personal social media accounts. The lookalike was known to one of the authors and judged to be a good match to the culprit by the authors. He provided photos from his social media account.

### Procedure

Participants were invited to take part in an online study. In the first session, after participants viewed the mock crime and completed the survey, they were assigned to one of three conditions. There were two experimental conditions (culprit; lookalike), and one control condition (see Table 1). Participants in the two experimental conditions viewed the simulated social media site, FriendFace. They were asked to imagine they had attended the fictitious event where the crime had taken place and were given the following instructions “You have just watched a video of a crime that took place during a social event. This event has a page on social media, which you can now view on the computer. Browse through the social media site and see if the person you saw stealing the handbag in the video appears on the site. You can take as long as you want to do this.” Participants were not asked whether or not they saw this person on the site at this stage. In one condition, the profile of the culprit was linked to the FriendFace site, and the participant could view images of the culprit. In the other condition, the lookalike's profile was linked to the site, and the culprit was not present. All participants saw the same culprit committing the crime, and those who saw a lookalike all saw the same lookalike of this culprit. Profiles of other potential “attendees” were also linked to the site. Participants were free to explore the site for as long as they wanted. Participants in the control condition did not view the FriendFace site but took part in the alternative activity.

The second session took place 7–8 days later and was triggered by an automated email. The delay was chosen to mimic a minimum forensically realistic delay. Participants viewed a photo lineup and were given the following instructions. “Last week you watched a video of a mock crime and browsed a social media page. Now you will see photos of people. Each photo will be numbered. The man you saw in the crime video may or may not be included. Please decide if you see the man from the crime video. If you see him, please write down what number he is. When they saw the lineup they were asked, “Is the person you saw in the crime video also in the lineup?” Participants could select “yes” or “no.” If they selected “yes” they were asked to provide the number of the individual that they thought was the culprit. All participants were then asked, “How sure do you feel that your answer is right on a 1–7 scale?” They responded from 1 (very unsure) to 7 (very sure). Some saw a lineup that contained the culprit (TP), while others saw a lineup that contained the lookalike (TA). They were asked to provide their identification decision and their confidence in that decision in the response boxes provided. 24% did not return for the second session, so their data were excluded from the final sample.

The experiment received approval from the Open University Research Ethics Committee (Reference: HREC/3264/Elphick).

## Results

### The Relationship Between Ethnicity and Accuracy

As participants of different ethnicities took part and the images were of white faces, we started by exploring whether the “other race effect” (e.g., Sporer, 2001) was reflected in the accuracy of the identification responses. We divided participants into those who identified as Caucasian (*n* = 171), and those who did not (*n* = 41) and found no difference in accuracy between the two ethnic categories, *X*^2^(1) = 0.002, *p* = 0.96, *V* = 0.003. Therefore, all participants were retained for subsequent analyses.

### The Relationship Between Time Spent on FriendFace and Accuracy

Participants in the experimental conditions (*n* = 232) spent between 144 and 2312 s looking at FriendFace (*M* = 447.22, *SD* = 240.33). Despite this large range, a logistic regression revealed that there was no statistical significance between time spent on FriendFace and lineup identification accuracy, *X*^2^(1) = 0.002, *p* = 0.97 (Inaccurate participants: *M* = 447.73; *SD* = 266.43, Accurate participants: *M* = 446.30; *SD* = 184.78). Longer search times were not associated with more accurate identifications. Therefore, all participants were retained for the following analyses.

### Target Present Accuracy

**Figure 1:**
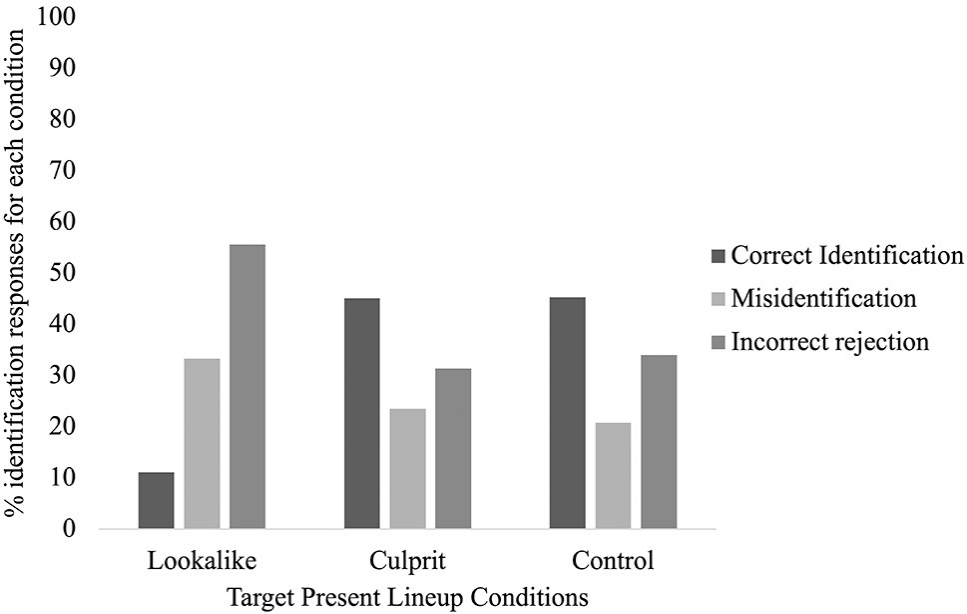
The proportion of identification responses (correct identifications, misidentifications, and incorrect rejections) for the TP lineup conditions (culprit, lookalike, and control).

In the TP condition, 34.9% of participants correctly identified the target, 25.5% misidentified a distractor, and 39.6% incorrectly rejected the faces. We explored relationships between *social media* (culprit; lookalike; control) and *identification response* (correct identification; misidentification; incorrect rejection). A Chi Square analysis revealed a significant relationship, *X*^2^(4) = 16.27, *p* = 0.01, *V* = 0.23 with a medium-large effect size. Inspection of Figure 1 reveals that this was driven by participants in the lookalike condition, as only 11.11% of them correctly identified the culprit (compared with 45.09% of participants in the culprit condition and 45.28% in the control condition). A considerably greater proportion of them made an incorrect rejection (55.55%) than participants in the other conditions (culprit condition = 31.37%; control condition = 33.96%). A greater proportion of them also made a misidentification (33.33%) than participants in the other conditions (culprit = 23.53%; control = 20.75%). Thus, in TP lineups, seeing a lookalike on social media negatively affected lineup accuracy, compared to either seeing the culprit, or not seeing the social media page at all.

### Target Absent Accuracy

**Figure 2:**
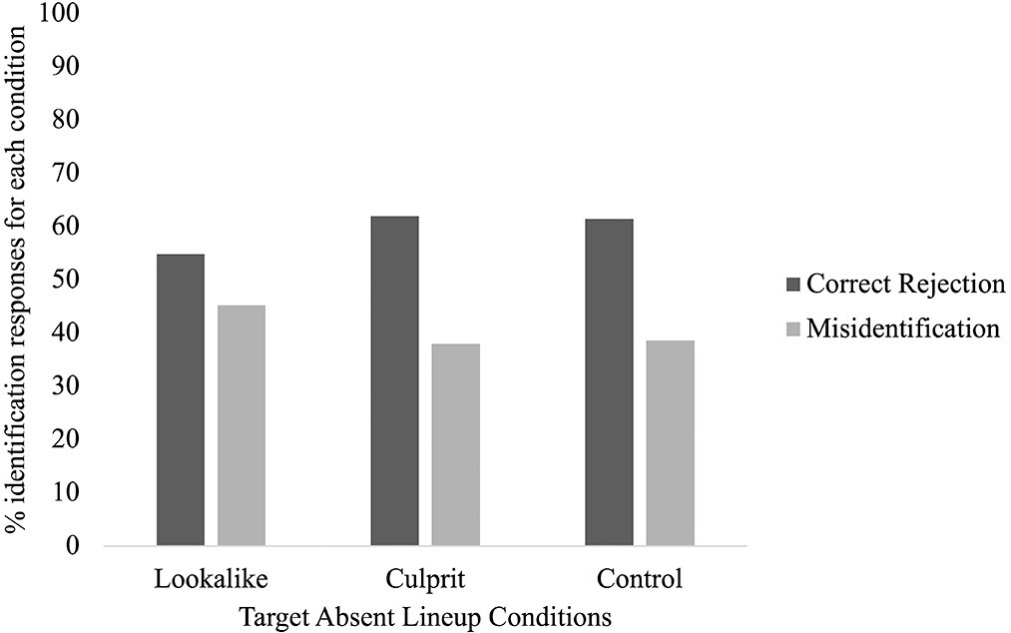
The proportion of identification responses (correct rejections, and misidentifications) for the target absent lineup conditions (culprit, lookalike, and control).

In the TA condition, 59.6% correctly rejected the faces and 40.4% misidentified a distractor. We explored relationships between *social media condition* (culprit; lookalike; control) and *identification response* (correct rejection; misidentification). However, we found no significant relationship, *X*^2^(2) = 0.58, *p* = 0.75, *V* = 0.07 (see Figure 2). Thus, it made no significant difference to lineup performance in TA lineups if participants saw either the culprit or a lookalike on social media or did not see social media at all. However, it is interesting to see from Figure 2 that the proportion of correct and incorrect responses in the culprit and control conditions are almost identical (62 and 61.36% were correct, respectively), while the pattern in the lookalike condition is slightly different (54.76% were correct).

**Figure 3:**
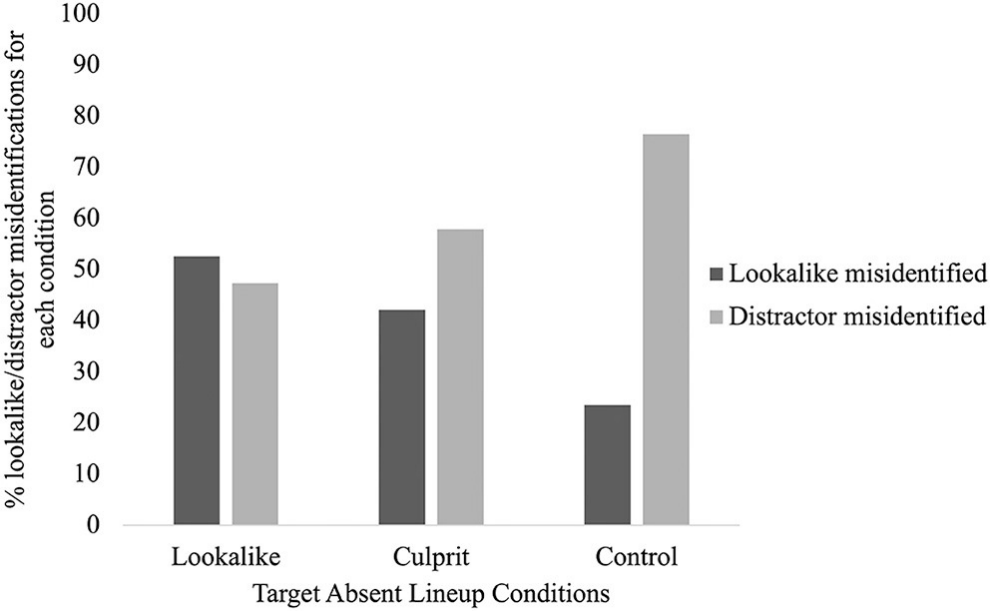
The proportion of lookalike and distractor misidentifications for the target absent lineup conditions (culprit, lookalike, and control).

When examining the proportion of lookalike misidentifications in each TA condition compared to misidentifications of another distractor, it was revealed that the lookalike was misidentified 40% of the time, and that participants in any one condition were not significantly more or less likely to misidentify the lookalike than in either of the other two conditions, *X*^2^(2) = 3.22, *p* = 0.20, *V* = 0.24. Despite this non-significant result, inspection of Figure 3 shows that only about quarter (23.53%) of control participants misidentified the lookalike, compared with 42.11% in the culprit condition and about half (52.63%) in the lookalike condition.

### Overall Accuracy

Comparing conditions by analyzing TP and TA lineups separately can be problematic as participants may adopt different thresholds for selection. For example, they may be more conservative in one condition meaning they are less likely to select someone from the lineup. In light of this, accuracy was also analyzed by computing the signal detection measures *d'* and *C* for each condition. For *d'*, which is a measure of sensitivity, this yielded values of 0.15 in the control condition, 0.23 in the culprit condition and −1.21 in the lookalike condition. Criterion (*C*) provides a measure of bias. Mean criterion in the control condition (−0.10) and the culprit condition (−0.11) indicates fairly balanced responding, with only a small liberal bias, while the positive criterion value in the lookalike condition (0.36) indicates a conservative bias. This again suggests that the difference in conditions is driven by poor performance in the lookalike condition, where (unlike the other two conditions) participants were more likely to make a misidentification than a correct identification.

### Confidence

The relationship between confidence and accuracy was examined using point-biserial correlations for “choosers,” which revealed a significant relationship, *r* (143) = 0.15, *p* < 0.05 (1-tailed), and “non-choosers,” where the relationship was non-significant, *r* (143) = 0.02, *p* = 0.84 (2-tailed). Noting the limitations of using point-biserial correlations, confidence-accuracy characteristic (CAC) analysis was conducted using the procedure described by Mickes (2015) for estimator variables, whereby foil choices from both TA and TP lineups are included. To balance N across different confidence levels, the 1–7 scale was collapsed into low (points 1–2), medium (points 3–4), and high (points 5–7).

**Figure 4:**
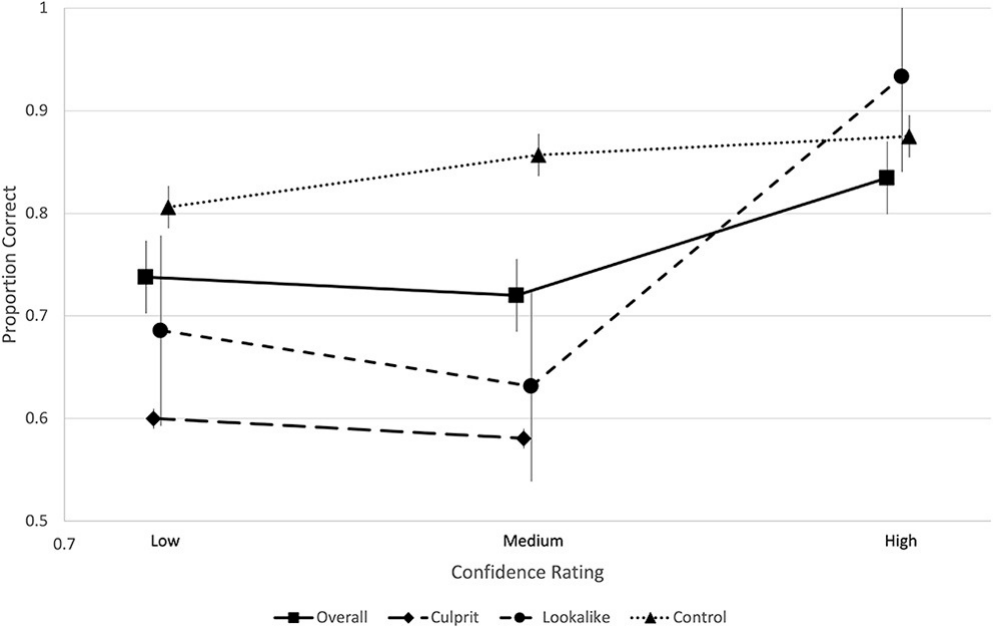
CAC analysis overall and by condition.

Figure 4 shows that participants overall (and in the control and culprit condition) used confidence (more or less) appropriately, particularly with high confidence being associated with a higher proportion of correct decisions. It was not possible to calculate the proportion correct scores associated with high confidence for the lookalike condition as no correct IDs were made with a high level of confidence.

## Discussion

The present study provides evidence that searching for a culprit on social media can negatively affect identification accuracy in TP lineups. Participants who saw a *lookalike* on social media after witnessing a crime were less likely to recognize the culprit in a subsequent lineup (compared with the other participants). The results indicate that if an eyewitness seeks a culprit on social media that contains a lookalike (between witnessing a crime and doing a lineup where the culprit is present), they are less likely to make a positive identification in the lineup. The research also explored the effect in TA lineups, but no significant relationship was found. The study indicated that the effects of reinforcing and conflicting post-event information were asymmetrical, as the *negative* effect on accuracy in TP lineups of seeing a lookalike during a social media search was not mirrored by a *positive* effect on accuracy of seeing the culprit.

The present research is the first to investigate the effect of self-directed social media searching on identification accuracy. The results support previous eyewitness research showing that mugshot exposure (Deffenbacher et al., 2006) and street identifications (Valentine et al., 2012; Davis et al., 2015) can bias subsequent identification accuracy, as seeing a lookalike on social media in the present study resulted in a greater proportion of errors in subsequent TP lineups. However, the finding that seeing a lookalike on social media reduces correct identification by over 30% is dramatic compared to other studies (Memon et al., 2011). Unlike some previous research (e.g., Davis et al., 2014), that has found composite construction leads to a higher rate of correct identifications compared to a control, our results were more similar to the majority of composite research (Pike G. E. et al., 2019; Pike G. et al., 2019; Tredoux et al., 2020) in finding no significant improvement in correct identifications between the Culprit and Control conditions.

Jenkins et al. (2011) study could offer some insight into our results. It highlighted the challenge of “telling [unfamiliar] people together” (taking into account differences seen in different images of an individual's face, due to lighting, viewpoint, etc.), rather than the challenge of “telling [unfamiliar] people apart.” These different challenges could shed light on the fact that social media exposure only affected *TP* lineup accuracy in the present research. In TA lineups, eyewitnesses only have to be able to “tell people apart,” whereas in TP lineups, they *also* have to be able to “tell people together” (they have to compare the culprit's face as they are viewing it with their memory of the culprit's face) when making their identification decision. Thus, not only do eyewitnesses have to be good at both tasks, but the additional task of “telling people together” could be the harder of the two (Jenkins et al., 2011).

A limitation is that we only used one culprit and one lookalike, so the effect could be attributed to a single item, an issue that could be usefully addressed in future research by using a larger sample of culprits and lookalikes and presenting them in different lineup positions. Another limitation is that we did not collect FriendFace identification data. This may have helped us to determine where any effect had occurred (e.g., on social media or during the lineup), and it might have influenced subsequent lineup response decisions (supporting the notion of a “commitment effect,” e.g., Godfrey and Clark, 2010; Valentine et al., 2012). It would be worth exploring this in future research by asking participants whether or not they thought they saw the culprit on FriendFace when they were still on the site. Also, the experiment emulated an eyewitness seeing a crime and conducting a self-directed search immediately. Despite the fact that the effect was medium-strong in the present research, it would be worth investigating whether the effect would be stronger if there was a delay between seeing the crime and conducting the self-directed search, as Paterson et al. (2009) found that post-event information had a greater effect when misinformation was presented after a delay (2 weeks rather than 20 min). It would be particularly useful to test this in TA lineups, as no significant effect was found in the present study.

The results presented here have implications for police and legal contexts, as they suggest that an eyewitness who searches social media and sees someone resembling the perpetrator may be more likely to make a misidentification than to correctly identify the culprit. In one sense this seems to support a fairly straightforward warning for criminal justice practitioners to be wary of an eyewitness who conducts their own investigation *via* social media. However, some care in this communication may be needed, given the tendency of law enforcement personnel to value positive lineup outcomes even if these occur at the expense of misidentifications (Pike et al., 2021). This would suggest that when disseminating the results to policing practitioners that it may be important to highlight the low hit rate and high incorrect rejection rate in the lookalike condition for target present lineups.

## Data Availability Statement

The raw data supporting the conclusions of this article will be made available by the authors, without undue reservation.

## Ethics Statement

The studies involving human participants were reviewed and approved by The Human Research Ethics Committee, The Open University. The patients/participants provided their written informed consent to participate in this study.

## Author Contributions

CE: conceptualization, methodology, verification, formal analysis, investigation, data collection, data curation, writing–original draft, writing–review and editing, visualization, and project administration. RP: conceptualization, methodology, formal analysis, and writing–review and editing. AStu, ZW, LF, and ML: conceptualization, methodology, and writing–review and editing. MZ: conceptualization and methodology. AStr: conceptualization, methodology, verification, resources, writing–review and editing, and supervision. CH: conceptualization, methodology, verification, resources, and writing–review and editing. GP: conceptualization, methodology, verification, resources, writing–review and editing, supervision, and funding acquisition. BP and BN: conceptualization, methodology, supervision, and funding acquisition. AB: conceptualization, methodology, writing–review and editing, supervision, and funding acquisition. All authors contributed to the article and approved the submitted version.

## Conflict of Interest

The authors declare that the research was conducted in the absence of any commercial or financial relationships that could be construed as a potential conflict of interest.

## Footnotes

^1^Undisclosed (n.d.). https://undisclosed-podcast.com.

^2^Crime Stoppers (n.d.). https://crimestoppers-uk.org.

^3^Vanished Podcast (n.d.). http://www.thevanishedpodcast.com.

^4^*State of Oregon v. Soza* [2008] WL 4455613.

^5^*R. v. McCullough* [2011] EWCA Crim 1413.

^6^*R v Alexander and McGill* [2012] EWCA Crim 2768.

^7^*R v Benfield* [2015] SADC 150.
