# Supplementary material for: Digital Detectives: Websleuthing Reduces Eyewitness Identification Accuracy in Police Lineups
Source: Front Psychol. 2021 Apr 15;12:640513. doi: 10.3389/fpsyg.2021.640513 (PMC8081957; doi:10.3389/fpsyg.2021.640513)
Supplement: Supplementary file 1 [file Data_Sheet_1.docx]

FriendFace Home Page with clickable Guest List thumbnails.

Example of a FriendFace profile. Participants could click through 3 images of each profile.

[Faces have been blacked out for this appendix]
